# Radiosynthesis and evaluation of an ^18^F–labeled silicon containing exendin-4 peptide as a PET probe for imaging insulinoma

**DOI:** 10.1186/s41181-017-0036-6

**Published:** 2018-01-02

**Authors:** Lukas O. Dialer, Andreas Jodal, Roger Schibli, Simon M. Ametamey, Martin Béhé

**Affiliations:** 1 Center for Radiopharmaceutical Sciences (CRS) of ETH, PSI and USZ, Institute of Pharmaceutical Sciences, Department of Chemistry and Applied Biosciences, ETH Hönggerberg, ETH Zurich, Zurich, Switzerland; 2 Center for Radiopharmaceutical Sciences (CRS), Research Department Biology and Chemistry, Paul Scherrer Institut, CH-5232 Villigen, Switzerland

**Keywords:** Pet, Insulinoma, Exendin-4, Silicon, ^18^F–Radiolabeling, Glp-1R

## Abstract

**Background:**

Analogues of exendin-4 have been radiolabeled for imaging the glucagon-like peptide type 1 receptors (GLP-1R) which are overexpressed in insulinoma. The aim of this research was to synthesize an ^18^F–labeled silicon containing exendin-4 peptide (^18^F-**2**) and to evaluate its in vitro and *in vivo* behavior in CHL-GLP-1 receptor positive tumor-bearing mice.

^18^F–labeled silicon containing exendin-4 peptide (^18^F-**2**) was prepared via one-step nucleophilic substitution of a silane precursor with ^18^F–fluoride in the presence of acetic acid and K222. ^18^F-**2** was then administered to tumor-bearing mice for PET imaging and *ex vivo* biodistribution experiments.

**Results:**

^18^F-**2** was produced in a radiochemical yield (decay corrected) of 1.5% and a molar activity of max. 16 GBq/μmol. The GLP-1R positive tumors were clearly visualized by PET imaging. Biodistribution studies showed reduced uptake of ^18^F-**2** in the kidneys compared to radiometal labeled exendin-4 derivatives. The radiotracer showed specific tumour uptake which remained steady over 2 h.

**Conclusions:**

This exendin-4 analogue, ^18^F-**2**, is a potential probe for imaging GLP-1R positive tumors.

## Background

β-cells are located within the islet of Langerhans in the pancreas beside α-, δ- and PP-cells. A function of β-cells is the blood sugar depended excretion of insulin (Vaidakis et al. 2010; Shin et al. 2010). Insulinoma are tumors originating from β-cells. Due to the independent secretion of insulin, patients with insulinoma may be hypoglycaemic and experience neuroglycopenic symptoms (Grant 1996; Boukhman et al. 1998; Service 1995; Grant 2005; Metz and Jensen 2008). Accurate localization of the usually benign lesions is essential for successful surgical excision, but suffers from a low sensitivity by the common diagnostic approaches. The invasive method of endoscopic ultrasound shows the highest diagnostic sensitivity with 85% whereas other non-invasive modalities such as transabdominal ultrasonography, CT, MRI or nuclear medicine modalities (somatostatin scintigraphy or ^18^F–DOPA) show a much lower sensitivity (Shin et al. 2010; Noone et al. 2005; Batcher et al. 2011; Finlayson and Clark 2004). In order to locate and to improve the preoperative planning and increase accuracy in surgery, new and more sensitive strategies are urgently needed (Noone et al. 2005; Tucker et al. 2006; Ramage et al. 2005).

The glucagon-like peptide type 1 receptor (GLP-1R) is overexpressed in virtually all benign insulinoma with high incidence and density and is considered a valuable target for the efficient visualisation by radiotracers (Reubi and Waser 2003; Wild et al. 2011; Wild et al. 2008; Korner et al. 2012). The natural ligand released from ileal L-cells, glucagon-like peptide 1 (GLP-1) stimulates the insulin secretion in β-cells by binding to the GLP-1R. Natural GLP-1 however shows a short metabolic half-life of less than 2 min due to degradation by the enzyme dipeptidyl peptidase IV (DPP4) (Pauly et al. 1999). Exendin-4, a 39-amino acid peptide found in the saliva of the Gila monster, has similar affinity and biological activity to GLP-1R while being metabolically resistant (Parkes et al. 2001). Analogues of exendin-4 have been labeled with ^125^I (Singh 1994), ^111^In (Wild et al. 2006; Gotthardt et al. 2006), ^99m^Tc (Wild et al. 2010), ^68^Ga (Wild et al. 2010), and more recently with ^18^F (Gao et al. 2011; Kiesewetter et al. 2012; Wang et al. 2012; Keliher et al. 2012; Kiesewetter et al. 2012; Wu et al. 2013). Several SPECT tracers based on exendin-4 were used in clinical studies with good results; however, they suffer from a high kidney uptake which hampers an optimal diagnosis and localisation. ^18^F–labeled exendin-4 analogues promise improved properties for clinical applications because of: (1) high sensitivity and high resolution images due to the low positron (*β* ^+^) energy (0.64 MeV), (2) reduced radiation burden for the patient and (3) the possibility to quantify.

In this study, we evaluated an exendin-4 analogue, modified with a silicon containing building block, in order to elucidate its potential as an imaging agent for targeting GLP-1R positive insulinoma. We and others have reported on the use of di-*tert*-butylphenylsilane building block for the direct one-step ^18^F–labeling of biomolecules (Mu et al. 2008; Hohne et al. 2008; Schirrmacher et al. 2006; Schirrmacher et al. 2007; Wangler et al. 2010; Iovkova et al. 2011; Schulz et al. 2011; Kostikov et al. 2012). Our group has shown that bombesin derivatives modified with this di-*tert*-butylphenylsilane building block are rather very lipophilic and are cleared predominantly via the hepatobiliary pathway (Hohne et al. 2008). Previous studies with radiolabeled exendin-4 derivatives have documented high kidney uptake, which potentially make the visualization of the pancreas difficult. We reasoned that by incorporating the rather highly lipophilic di-*tert*-butylphenylsilane building block in exendin-4, we could significantly reduce the kidney uptake and thereby shift renal to hepatobiliary clearance. Herein we report on the radiosynthesis, in vitro and in vivo evaluation of a ^18^F–silicon-based exendin-4 derivative as a probe for imaging GLP-1R positive insulinoma.

## Methods

### General

The reagents and solvents were purchased from Sigma-Aldrich Chemie GmbH, Fluka Chemie AG, Archimica GmBH, Chemie Brunschwig AG, Acros Organics, ABCR GmbH & Co. or VWR International AG and were used as supplied unless stated otherwise. Analytical high-performance liquid chromatography (HPLC) was performed with a reversed-phase column (ACE C18, 50 × 4.6 mm, 5 μm). Semi-preparative radio-HPLC purification was performed with a reversed-phase column (ACE C18, 250 × 10 mm, 5 μm). Both analytical and semi-preparative HPLC chromatograms were obtained by use of an Agilent 1100 system equipped with multi-UV-wavelength (measuremt were done at 254 nm) and Raytest Gabi Star detectors and Gina Star software.

### Chemistry

*His-Gly-Glu-Gly-Thr-Phe-Thr-Ser-Asp-Leu-Ser-Lys-Gln-Nle-Glu-Glu-Glu-Ala-Val-Arg-Leu-Phe-Ile-Glu-Trp-Leu-Lys-Asn-Gly-Gly-Pro-Ser-Ser-Pro-Ala-Pro-Pro-Pro-Ser-Lys-(N* ^*6*^ *–2-(4-(di-tert-butylsilyl)phenyl)acetyl)-NH*_*2*_ *(precursor* ***1*** *).* Precursor **1** was provided by Peptide Specialty Laboratories GmbH, Heidelberg, Germany as lyophilized white solid. The product was re-analyzed by the MS-Service at LOC of ETH Zurich. HRMS (ESI-MALDI) calcd. For [C_207_H_321_N_52_O_62_Si]^+^: 4555.3328, found: 4555.3496. The purity (> 95%) of precursor **1** was confirmed by analytical HPLC (gradient acetonitrile/H_2_O + 0.1% TFA 5:95–95:5 in 20 min, 1.0 mL/min; R_T_ = 11.40 min).

*His-Gly-Glu-Gly-Thr-Phe-Thr-Ser-Asp-Leu-Ser-Lys-Gln-Nle-Glu-Glu-Glu-Ala-Val-Arg-Leu-Phe-Ile-Glu-Trp-Leu-Lys-Asn-Gly-Gly-Pro-Ser-Ser-Pro-Ala-Pro-Pro-Pro-Ser-Lys-(N* ^*6*^ *–2-(4-(di-tert-butylfluorosilyl)phenyl)acetyl)-NH*_*2*_ *(reference (Shin* et al. 2010 *)).* Precursor **1** (1.0 mg, 0.22 μmol) was dissolved in DMSO (200 μL). This solution was added to a mixture of KF (0.1 mg, 1.8 μmol), Kryptofix 222 (K222, 0.66 mg, 1.8 μmol), and K_2_CO_3_ (0.1 mg, 0.88 μmol). Glacial acetic acid (7.8 μL, 174 μmol) was added and the resulting suspension was heated at 70 °C for 30 min. An aliquot of the reaction mixture was purified by analytical HPLC (gradient acetonitrile/H_2_O + 0.1% TFA 5:95–95:5 in 20 min, 1.0 mL/min) to afford **2** (R_T_ = 11.18 min). The product was analyzed by LC-MS: *m*/*z* calcd.: 4573.3, found: 4573.0 ([M + H]^+^).

### Radiolabeling and hydrolytic stability studies

No-carrier-added [^18^F]fluoride was produced on an IBA Cyclone 18/9 cyclotron by irradiation of 98% enriched [^18^O]H_2_O (2.0 mL) using an 18-MeV proton beam via the [^18^O(p,n)^18^F] nuclear reaction. [^18^F]Fluoride was trapped on a preconditioned anion-exchange resin cartridge (Sep-Pak QMA Light; Waters; preconditioning with 0.5 M K_2_CO_3_-solution (5 mL), water (10 mL) and air (10 mL)). The cartridge was eluted with a solution of K222 (5.0 mg) and K_2_CO_3_ (1.0 mg) in acetonitrile (1.2 mL) and water (0.3 mL). The fluoride was dried by azeotropic distillation of acetonitrile at 100 °C under vacuum with a stream of nitrogen. The azeotropic drying process was repeated three times with acetonitrile (1 mL).

The radiosynthesis was performed manually in a hot cell using a manipulator. A solution of precursor **1** (4.0 mg, 878 nmol) and glacial acetic acid (10 μL) in anhydrous DMSO (150 μL) was added to the dry K[^18^F]F/K222 complex (typically 26–28 GBq) and heated at 110 °C for 15 min. After cooling at room temperature for 5 min, a mixture of acetonitrile/H_2_O + 0.1% TFA (2.0 mL, 1:1) was added to the reaction vial and the diluted mixture was purified by semi-preparative radio-HPLC using 0.1% TFA/water solution (solvent A) and 0.1% TFA/acetonitrile (solvent B) as the solvent system at a flow rate of 4 mL/min and with a gradient as follows: 0–15 min 95% A, 15–40 min 55% A. The fraction containing ^18^F-**2** was collected into a solution of 1 mM glutamic acid and 0.5% TFA/water solution (30 mL) and immobilized on a C18 cartridge (Sep-Pak Light C18, Waters, or Chromafix C18 (s), Machery-Nagel). After washing with 0.9% NaCl-water solution (20 mL), ^18^F-**2** (180–270 MBq, 1.0–1.5% dc RCY) was eluted with a solution of 1 mM HCl/ethanol (1 mL, 1:9) into a vial containing 0.15 M PBS solution (0.5 mL). The mixture was neutralized by adding a 1 mM NaOH aqueous solution (100 μL) and the ethanol was evaporated at 95 °C with a gentle stream of nitrogen. The identity of ^18^F-**2** was confirmed by comparison with the HPLC retention time of the nonradioactive reference compounds (R_T_ = 11.19 min) using analytical radio-HPLC (gradient acetonitrile/H_2_O + 0.1% TFA 5:95–95:5 in 20 min, 1.0 mL/min). For in vivo applications, ^18^F-**2** was passed through a sterile filter into a sterile, pyrogen-free vial. Hydrolytic stability of ^18^F-**2** was tested after the addition of PBS to an aliquot of the ethanolic solution of the product. Typical concentration of tracer was 30 MBq in 100–120 μL. The concentration of ethanol was equal or less than 5% providing a maximal ratio of EtOH:PBS of 1:19. This mixture was analyzed by HPLC at different time points.

### In vitro receptor binding assay

The binding affinities to the GLP-1 receptor for both the precursor compound **1** and native exendin-4 were determined using a displacement assay on CHL cells stably transfected with the GLP-1 receptor gene (van Eyll et al. 1994) grown in 6 well plates (0.8 × 10^−6^ cells/well grown overnight) at 90–95% confluence. The test compound solutions and [^125^I]-exendin-4 (9–39) (PerkinElmer, molar activity: 81.4 TBq/mmol, 100 μL, 0.9 pmol) were added to all well plates. The final concentrations of the test compound in the wells were in the range of 1 pM to 1 μM. For the total binding no cold peptide was added. The total volume was adjusted with medium containing 0.1% BSA to 1 mL. The cells were incubated at 4 °C for 1 h and subsequently washed twice with cold phosphate buffered saline (PBS) and solubilised with 0.5 mL 1 M NaOH (2×). The radioactivity was measured in a γ-counter (Packard Cobra II Auto Gamma, Perkin Elmer). The 50% inhibitory concentration (IC_50_) values were calculated using GraphPad Prism (GraphPad Software, La Jolla, CA) fitting the data with nonlinear regression using least squares fit. Experiments were performed on triplicate samples and repeated three times.

### Log D_7.4_ measurement

The determination of distribution coefficient (log D_7.4_) was carried out by the shake-flask method in analogy to a published procedure (Fischer et al. 2012). Briefly, 800 kBq ^18^F-**2** was added to a mixture of PBS (0.5 mL, pH = 7.4) and 1–octanol (0.5 mL) at room temperature. The mixture was equilibrated for 15 min in an overhead shaker and further centrifuged (3 min, 5000 rpm). Aliquots (50 μL) of both phases were analyzed in a γ–counter (1480 Wizard, PerkinElmer). The partition coefficient was expressed as the ratio between the radioactivity concentrations (cpm/mL) of the 1–octanol and the PBS phase. Values represent the mean ± standard deviation of five determinations from one experiment.

### Animals

Animal studies complied with Swiss laws on animal protection and husbandry and were approved by the Veterinary office of the Canton Zurich. After an acclimatization period, tumor xenografts were produced in 6-week old female CD1 nu/nu mice by subcutaneous injection in both shoulder regions of CHL-GLP-1 receptor positive cells (8 × 10^6^ cells/mouse in PBS (100 μL, pH 7.4)) under 2%–3% isoflurane anaesthesia. *Ex vivo* biodistribution experiments were conducted three weeks after the inoculation. PET imaging experiments were performed five weeks after the inoculation.

### Ex vivo biodistribution

Tumor-bearing mice (*n* = 12) were injected intravenously with ^18^F-**2** (200 kBq in 100 μL PBS, 1.3 pmol, 5.9 ng). The animals were sacrificed at 30 min, 1 h and 2 h post injection (*n* = 4 for each time point). To determine specific binding an additional group of tumor-bearing mice (*n* = 4) received nonradioactive precursor **1** (100 μg in 100 μL PBS, 22 nmol) co-injected with ^18^F-**2** (266 kBq in 100 μL PBS, 1.7 pmol, 7.8 ng) and were sacrificed 1 h post injection. Organs and tissues of interest were collected and weighed, and the amount of radioactivity was determined in a *γ*-counter (1480 Wizard, PerkinElmer) to calculate percentage uptake (% injected dose per gram of tissue). Statistical significance was calculated using Student *t*-test (two populations, unpaired). *P* values of less than 0.05 were considered statistically significant.

### Small animal PET/CT

Two tumor bearing mice were injected with ^18^F-**2** (13.0 MBq in 80 and 100 μL PBS containing ≤ 5% ethanol, 1.3 and 1.9 nmol) via lateral tail vein. Anaesthesia was induced with 5% isoflurane (Abbott) in O_2_/air 5 min before PET/CT acquisition. Depth of anaesthesia and temperature were controlled as described by Honer et al. (Honer et al. 2004). PET/CT scans were performed under 2–3% isoflurane anaesthesia with a GE VISTA eXplore PET/CT tomograph. Static scans were carried out 120–150 min p.i. in two bed positions (15 min upper body followed by 15 min lower body) with tumor-bearing mice. Data were reconstructed by two-dimensional ordered-subset expectation maximization (2D OSEM). Region of interest analysis was conducted with the PMOD 3.3 software (PMOD, Switzerland). The xenograft, kidney and liver volumes of interest were drawn according to the PET/CT images and average background activity was estimated from a sphere with a volume of ca. 0.5 cm^3^ between the shoulder regions. Standardized uptake values (SUV) were calculated as a ratio of tissue radioactivity concentration (kBq/cm^3^) and injected activity dose per gram body weight (kBq/g) at the scan start. Percentage injected dose per gram of tissue (%ID/g) was calculated using SUV values: SUV / body weight [g] × 100% = %ID/g.

## Results

### Radiolabeling, hydrolytic stability studies and log D_7.4_ measurement

**Fig. 1:**
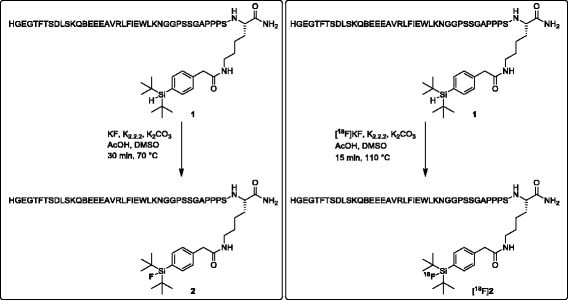
Synthesis of reference peptide **2** and ^18^F–labeling of [^18^F]**2**

^18^F–Labeling of silicon containing exendin-4 analogue proceeded as depicted in Fig. 1. The synthesis started typically with 26–28 GBq of the dried K[^18^F]F/K222 complex and was finished within 60–75 min and maximal ^18^F–incorporation yield was 6%. After HPLC purification and formulation 180–270 MBq of ^18^F-**2** were obtained. The radiochemical yield was 1.0–1.5% (decay corrected) and [^18^F]**2** was afforded in ≥ 95% radiochemical purity and molar activity of 12–16 GBq/μmol (end of synthesis). The hydrolytic stability test of ^18^F-**2** in PBS showed that no defluorination occurred within 2 h (data not shown). The log D_7.4_ value of ^18^F-**2 **as a measure of hydrophilicity was determined by the shake-flask method and amounted to −1.38 ± 0.06 (*n* = 5), indicating good water solubility.

### In vitro receptor binding assay

The IC_50_ value determined for precursor **1** was 169 ± 19 nM, which is approximately fifteen-fold higher compared to exendin-4 (10.4 nM ± 1.6 nM).

### Ex vivo biodistribution

Table 1 summarizes the *ex vivo* biodistribution data of ^18^F-**2** in xenograft-bearing mice. Thirty minutes after injection, tumor uptake was 15 ± 7%ID/g. It essentially remained steady over the measured period of time and was reduced significantly by a blocking dose of precursor **1** (*P* < 0.03). Tracer uptake in the lungs was constant and in the same range as in the tumors. Radioactivity uptake in the pancreas and the stomach was lower and decreased slightly over time. The GLP-1 receptor-positive organs lung and stomach revealed a significantly reduced tracer uptake under blocking conditions. Tracer accumulation in blood was high (9.7 ± 1.1%ID/g) at 30 min p.i. and decreased after 120 min p.i. (3.3 ± 0.8%ID/g), resulting in increased tumor to blood ratio (4 ± 2 at 120 min p.i.), but indicating a relatively slow blood clearance of the tracer. Bone uptake remained steady at ~ 2%ID/g and highest accumulation of the radiotracer was found in kidneys (49 ± 18%ID/g at 60 min p.i.).

**Table 1:** Biodistribution data of [^18^F]**2** in nude mice bearing CHL-GLP-1 receptor positive tumor xenografts

Organ or tissue	30 min p.i. (*n* = 4)	60 min p.i. (*n* = 4)	60 min p.i. (*n* = 4) blockade	120 min p.i. (*n* = 4)
%ID/g in				
Blood	9.7 ± 1.1	6.9 ± 2.2	11.1 ± 0.6	3.3 ± 0.8
Lungs^a^	15.1 ± 1.5	15 ± 4	10.0 ± 0.4	11.4 ± 2.1
Spleen	2.9 ± 0.3	2.4 ± 0.8	3.5 ± 0.1	1.6 ± 0.5
Kidneys	33.3 ± 2.4	49 ± 18	79 ± 5	39 ± 12
Pancreas^a^	4.2 ± 0.6	4.4 ± 1.4	6 ± 4	3.2 ± 0.6
Stomach^a^	1.8 ± 0.7	1.3 ± 0.2	1.0 ± 0.1	1.0 ± 0.7
Intestines	3.0 ± 0.3	5.4 ± 0.8	5.3 ± 1.2	7 ± 4
Liver	6.4 ± 0.7	7.7 ± 2.1	10.60 ± 0.30	5.0 ± 1.1
Muscle	1.5 ± 0.4	1.1 ± 0.4	1.73 ± 0.13	0.61 ± 0.12
Bone	1.8 ± 0.3	2.0 ± 0.7	2.87 ± 0.20	1.9 ± 0.5
Tumor^a^	15 ± 7	14 ± 7*	7 ± 1*	13 ± 10

Mice were injected with [^18^F]**2** (200 kBq, 1.3 pmol) via the lateral tail vein. In the blockade group, each animal received nonradioactive precursor **1** (100 μg, 22 nmol) in PBS co-injected with tracer [^18^F]**2** (266 kBq, 1.7 pmol)

*Values are significantly different (unpaired, two populations, Student t-test, *P* < 0.03)

^a^GLP-1 receptor-positive organs

### Small-animal PET/CT

**Fig. 2:**
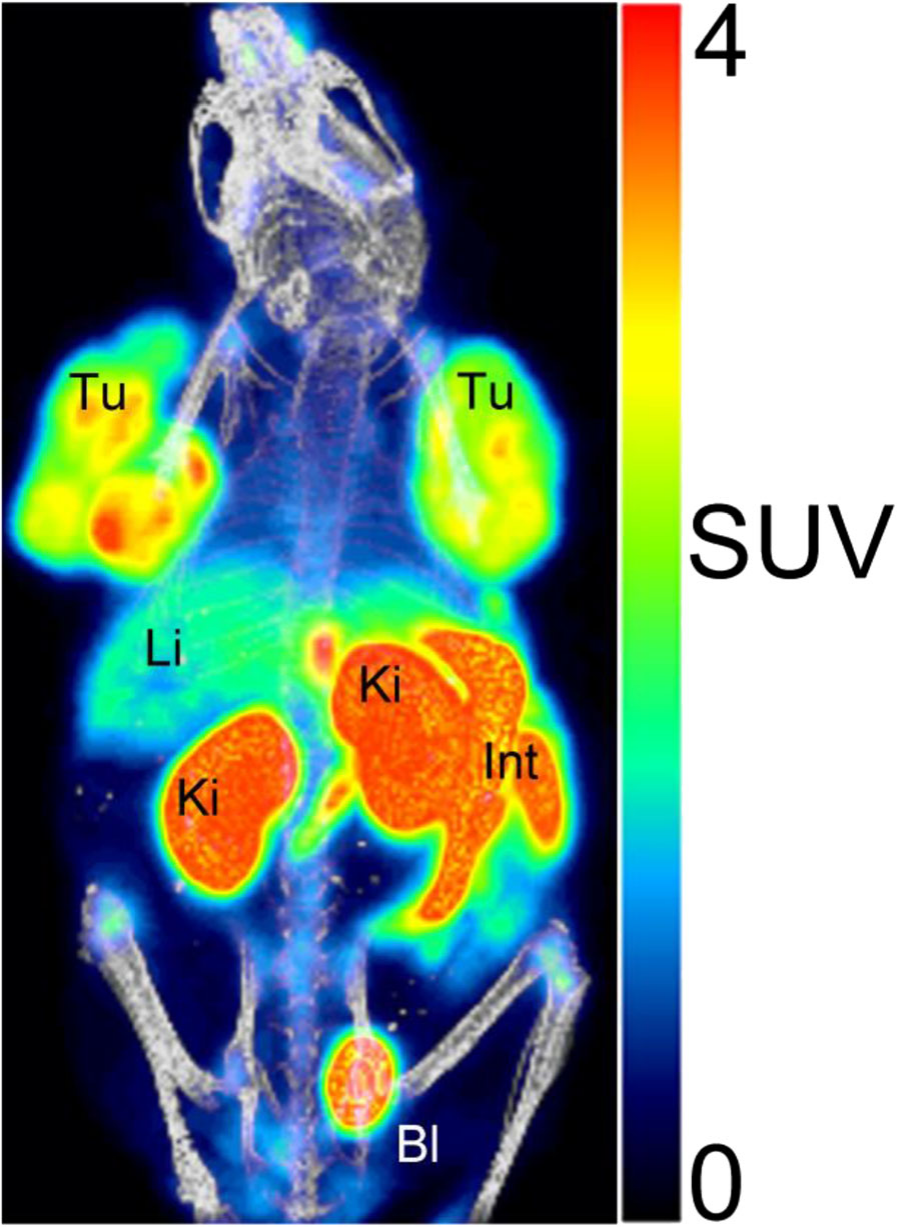
PET/CT image (three-dimensional, maximum intensity projections (MIP)) of a female CD1 nu/nu mouse. Static scan (whole body, 120–150 min p.i.) of CHL-GLP-1 tumor-bearing mouse injected with [^18^F]**2** (12.7 MBq (1.3 nmol)). Anesthesia was maintained with 2–3% isoflurane in O_2_/air. *SUV* standardized uptake value, *Tu* tumor, *Ki* kidneys; *Li* liver, *Bl* urinary bladder, *Int* intestinal tract

A PET/CT image of a tumor bearing mouse after injection of ^18^F-**2** is shown in Fig. 2. The highest radioactivity concentrations were observed in the kidneys, intestine, and urinary bladder, whereas the tumors are more visible than the liver and bones. Highest uptake from 120 to 150 min p.i. of ^18^F-**2** was in the kidneys (SUV_kidneys_: 4.1 and 5.0) followed by the tumors (SUV_tumors_: 2.2 and 2.3) and liver (SUV_liver_: 1.4 and 1.6). Tumor to background ratio was ~ 3.

## Discussion

It was shown by different groups that radiolabeled exendin derivatives are good tracers for targeting the GLP-1R (Wild et al. 2010). The clinical application of exendin tracers is limited to SPECT tracers with ^99m^Tc and ^111^In being the most widely used radionuclides (Wild et al. 2008; Pach et al. 2013). The use of a PET tracer has the advantages of higher sensitivity, better resolution and also the possibility to quantify. The most frequently used PET radionuclide is ^18^F–fluorine. It is widely available and has good physical properties (e.g. low *β* ^*+*^-energy of 0.64 MeV). Therefore, the development of ^18^F–exendin tracer would have a high impact in imaging β-cell derived diseases.

The silicon-based building block can be used for a direct one-step ^18^F–fluorination of biomolecules, as was shown with bombesin analogues, octreotate derivatives and other biomolecules (Mu et al. 2008; Hohne et al. 2008; Schirrmacher et al. 2006; Schirrmacher et al. 2007; Wangler et al. 2010; Iovkova et al. 2011; Schulz et al. 2011; Kostikov et al. 2012). The radiolabeling reaction conditions are compatible with peptides and the ^18^F–fluoride–silicon bond of the di-*tert*-butyl silyl building block has been shown to be hydrolytically stable against defluorination under physiological conditions (Hohne et al. 2008). We applied the procedure established in our laboratory (Hohne et al. 2008) to successfully synthesize ^18^F-**2**in a one-step reaction via nucleophilic substitution of a silane precursor with ^18^F–fluoride in the presence of acetic acid and K222. The final compound was obtained within 60–75 min, in good radiochemical purity, and in sufficient amount for in vivo studies. To our knowledge, exendin-4 analogue ^18^F-**2**, a forty amino acid peptide, is currently the largest peptide containing an organosilicon moiety that has been labeled with ^18^F in one step by nucleophilic substitution. Highest ^18^F–incorporation (6%) was achieved by using 4 mg (878 nmol) of the silane precursor. The labeling reaction mixture contained unidentified side-products, which were easily removed by HPLC purification, however, for ^18^F-**2** and precursor **1** no clear-cut baseline separation was achieved. The consequence of this was that some amount of the silane precursor **1** was still found in the formulated product solution, which partly contributed to the rather low molar activity of 12–16 GBq/μmol.

^18^F-**2** is a hydrophilic peptide with a logD_7.4_ value of −1.38 ± 0.06, however compared to other radiolabeled exendin derivatives (Keliher et al. 2012), it is more lipophilic, which is not unexpected due to the bulky lipophilic silicon-based building block. Besides the high lipophilicity, this moiety negatively impacted on the receptor binding affinity (IC_50_). A fifteen-fold lower affinity value was obtained with **1** compared to parent exendin-4.

For *ex vivo* biodistribution, the highest tumor uptake of 15%ID/g was observed at 30 min p.i. Beyond this time point tumor retention was still high and amounted to 13%ID/g at 2 h p.i. The uptake in the kidneys was in the range between 30 to 50%ID/g which was dramatically lower compared to the kidney uptake of radiometal labeled compounds, which is normally much higher than 100%ID/g (Gotthardt et al. 2006; Brom et al. 2010). In turn, the liver uptake (6.4%ID/g at 30 min p.i. and 5.0%ID/g at 2 h p.i., respectively) was higher, as expected from the increased lipophilicity of ^18^F-**2**.

*Ex vivo* biodistribution data showed comparable uptake of the radiotracer in tumors and GLP-1 receptor-positive lungs at all examined time points. This finding was not confirmed by the PET image, which showed a high tumor to lungs contrast. This discrepancy can be explained by the different amounts of injected peptide (1.3–1.9 nmol) during the PET studies and during the ex vivo biodistribution studies (1.3 pmol). Since more unlabeled peptide was injected during the PET studies, the GLP-1 receptors in the lungs might have been saturated. The tumor uptake could significantly be decreased by co-injection of unlabeled exendin-4, suggesting specific binding of the radioligand to GLP-1 receptors.

An important aspect is the kidney uptake which is very high with the radiometal labeled exendin derivatives (Jodal et al. 2015; Jodal et al. 2017). We observed a dramatic reduction with our compound but with a comparable uptake in receptor positive tissues. This observation could be confirmed with other fluorine-18 labeling methods for exendin (Wu et al. 2013; Yue et al. 2014; Mikkola et al. 2016), with the exception of the radiolabeling method involving ^18^F–Al-NOTA, where the kidney uptake stays high (Xu et al. 2015; Mi et al. 2016). The conclusion out of this observation is that the inclusion of a radiometal has a negative impact on kidney uptake. In conclusion, we have successfully radiolabeled an exendin-4 containing forty amino acids using a single step and without a prosthetic group. The new exendin-4 derivative showed the expected biodistribution with a significantly lower kidney uptake compared to radiometal labeled exendin-4 derivatives. Specificity of binding to GLP-1R was also demonstrated. These results show that [^18^F]**2** may potentially find application in imaging insulinoma.

## Conclusion

We could show that the Exendin-4 can be labelled with ^18^F with the silicon based method retaining the main biological properties like affinity and exhibit a favourable biodistrution with a specific uptake in the receptor positive pancreas and tumor. The kidney uptake is markantly reduced compared to radiometal labelled Exendin-4 derivatives.

## Abbreviations

^18^F–DOPA: ^18^F–dihydroxyphenylalanine
DPP4: Dipeptidyl peptidase IV
GLP-1: Glucagon-like peptide 1
GLP-1R: Glucagon-like peptide type 1 receptor
HPLC: High-performance liquid chromatography
SUV: Standardized uptake value
